# Assessing the suitability of self-healing rubber glove for safe handling of pesticides

**DOI:** 10.1038/s41598-022-08129-9

**Published:** 2022-03-11

**Authors:** Janarthanan Supramaniam, Darren Yi Sern Low, See Kiat Wong, Bey Hing Goh, Bey Fen Leo, Siah Ying Tang

**Affiliations:** 1 Chemical Engineering Discipline, School of Engineering, Monash University Malaysia, 47500 Bandar Sunway, Selangor Darul Ehsan Malaysia; 2 Biofunctional Molecule Exploratory Research Group, School of Pharmacy, Monash University Malaysia, 47500 Bandar Sunway, Selangor Darul Ehsan Malaysia; 3 College of Pharmaceutical Sciences, Zhejiang University, Hangzhou, 310058 Zhejiang China; 4 Nanotechnology and Catalysis Research Centre, University of Malaya, 50603 Kuala Lumpur, Malaysia; 5 Department of Molecular Medicine, Faculty of Medicine, University of Malaya, 50603 Kuala Lumpur, Malaysia; 6 Advanced Engineering Platform, School of Engineering, Monash University Malaysia, 47500 Bandar Sunway, Selangor Darul Ehsan Malaysia

**Keywords:** Occupational health, Polymers, Chemical safety

## Abstract

Rubber gloves used for protection against chemicals or hazards are generally prone to tearing or leaking after repeated use, exposing the worker to potentially hazardous agents. Self-healing technology promises increased product durability and shelf life appears to be a feasible solution to address these issues. Herein, we aimed to fabricate a novel epoxidized natural rubber-based self-healable glove (SH glove) and investigate its suitability for handling pesticides safely. In this study, breakthrough time analysis and surface morphological observation were performed to determine the SH glove’s ability to withstand dangerous chemicals. The chemical resistance performance of the fabricated SH glove was compared against four different types of commercial gloves at different temperatures. Using malathion as a model pesticide, the results showed that the SH glove presented chemical resistance ability comparable to those gloves made with nitrile and NR latex at room temperature and 37 °C. The self-healing test revealed that the SH glove could be self-healed and retained its chemical resistance ability close to its pre-cut value. Our findings suggested that the developed SH glove with proven chemical resistance capability could be a new suitable safety glove for effectively handling pesticides and reducing glove waste generation.

## Introduction

Pesticide application in the agricultural sector has been increasing dramatically due to the rising demands of crops for food production. The continuous utilization of pesticides is encouraged by the strong perception that pesticides are highly effective in producing good quality crops, but adversely, they can be harmful to health and the environment^1,2^. Annually, there are 385 million cases of acute pesticide poisoning among agricultural workers reported worldwide^3^. Dermal contact is one of the most common routes of occupational exposure to pesticides which usually occurs when performing handling tasks such as diluting concentrated chemicals, during spraying and reentry works^4–6^. Positively, though, there is a general awareness among agricultural workers on the importance of using gloves as personal protective equipment (PPE) against dermal exposure to harmful pesticides. A chemical breakthrough occurs even the glove material was considered suitable for chemical handling^7^. Therefore, a glove is chemical resistant to confirm at least Class 2 protection index or breakthrough time > 30 min for the tested chemicals^8^.

A study conducted by Jomichen et al.^9^ on pesticide exposure from the Australian agricultural workforce reported that 70% of workers depend on gloves to prevent dermal exposure, with the majority of workers donning rubber gloves during pesticide handling. Gloves made from natural rubber (NR) latex are compatible with salts, ketones, alkaline and acidic working environments. In contrast, nitrile gloves are more suitable for handling alcohols, greases, acids, oils, and caustics^7^. According to a survey conducted by Hojerová et al.^10^ regarding the practice of protective glove usage among agricultural workers, more than 50% of workers reuse gloves over extended periods. The polymeric surface of the glove is the only natural barrier between the skin and the exterior environment of harmful materials. This repeated usage could lead to (micro)cracking or tears that would compromise the functionality of the glove and shorten its lifespan. A recent study conducted by Liem et al.^11^ on agricultural worker's exposure to pesticides in Central Java, Indonesia, found cases of workers who use chemical-resistant gloves often continue using the same pair of gloves even they are damaged. Furthermore, it was also found that workers from other industries such as in the cleaning sector and waste disposal continue to use damaged gloves as they are often not replaced in the event of minor tears^12–16^.

It is unlikely that small industries or ordinary gardeners would frequently replace reusable chemical-resistant gloves, owing to their relatively high cost and unawareness of the potential health impacts^17^. On the other hand, rapid disposal of gloves daily for slight tears would potentially increase waste generation. According to the recently published reports, gloves are one of the most common PPE pollution found in natural and urban environments^18–21^. Therefore, there is a need to innovate a rubber glove that could restore damages upon repeated use and extend its lifetime to reduce waste generation. Utilizing self-healing polymeric materials to fabricate reusable gloves is a potential solution for the problems. A self-healable material is typically characterized by its ability to recover from physical damages or cracks^22^. Random polymeric network structures can form new bonds in a self-healing system when old molecular bonds are broken. This fascinating technology has been explored in various literature sources^23–29^. Herein, we develop a lab-scale self-healing protective rubber glove using a modified conventional dipping method and determine its suitability for chemical handling. To our knowledge, no self-healing rubber glove was fabricated and tested so far for protective purposes against a highly concentrated pesticide.

In this study, a thermally responsive polymer with self-healing characteristics—a composite of epoxidized natural rubber (ENR) reinforced with ZnO modified cellulose nanofiber (ZnO-CNF) was selected to fabricate the SH glove. The absence of covalent crosslinks and the presence of excessive non-covalent networks exhibited destitute tensile strength in self-healing rubbers^30,31^. The tensile strength of self-healing rubber is often noticeably lower than 1 MPa^28,32^. Nanofillers such as talc, silica and carbon black are commonly used reinforcing agents to improve the strength and elasticity of the rubber composite^33,34^. Carbon black and silica are two most common reinforcing fillers used in rubber industry^35^. The use of these fillers is often associated with disadvantages such as utilization of non-renewable resources, energy-intensive manufacturing, lack of decomposability and greenhouse gas emission issues due to composite destruction by combustion^36–39^. Therefore, the demand for safe and less harmful reinforcing agents increases. Nanocellulose isolated plants and bacteria sources are potential rubber reinforcing agents due to their beneficial properties such as being, non-toxic abundantly available, sustainable, and renewable^40,41^. However, nanocellulose often suffers from agglomeration and weak interfacial adhesion on rubber matrix thus limiting the reinforcing efficiency^36,42^. To overcome this problem, the nanocellulose surface was chemically functionalized using ZnO for improved interaction with rubber matrix^42,43^. ZnO are highly stable nanoparticles and are common materials used in rubber vulcanization systems^44^. The self-healable rubber fabrication was based on a reaction between oxygenous groups along the ENR chain and the ZnO-CNF nanofiller, where Zn^2+^ interacts with ENR to form a cross-linked network. The role of cellulose in this study was to serve as a sustainable reinforcing agent in the rubber matrix to replace the conventional fillers such as carbon black and silica.

In this study, thermally responsive self-healing epoxidized natural rubber (ENR) reinforced with ZnO modified cellulose nanofiber (ZnO-CNF) was used to fabricate a SH glove. The chemical resistance properties of the SH glove after being exposed to the pesticide were evaluated via permeation breakthrough time analysis. Laboratory stimulated surface damage (minor cuts with scissors) was introduced to the tested gloves to reflect possible changes in the working environment. Four commercially available gloves were tested to benchmark the SH glove fabricated in this study. All the gloves were tested against a highly concentrated pesticide, before the damage, with damage and after damage recovery. The SH glove was tested against malathion, a widely used chemical in pesticide formulation^1^. The finding confirms the suitability of using a self-healing polymer to fabricate a protective glove for chemical handling. Our work not only offers an innovative route to extend the lifetime of a reusable glove but also address the environmental sustainability issue of glove use.

## Results

### Synthesis of the self-healing material

Firstly, the ZnO-CNF nanofiller was fabricated using the ultrasonic-assisted co-precipitation technique. The CNF surface changed to a slightly negative charge when dispersed in water, aided by hydroxyl group ionization^45^. The precursor (NaOH) reacted with the inactive cellulose hydroxyl group (Cell-OH) and produced base-activated cellulose (Cell-O^−^)^45,46^. The Zn^2+^ ions were embedded on the CNF surface via electrostatic interactions between the zinc cations and oxygen atoms of the polar hydroxyl groups of CNF^45,47^. Ionization in cellulose chains increased and promoted the ZnO nucleus formation^48–50^.

After loading ZnO-CNF to the ENR latex, the oxygenous groups of ENR reacted with ZnO-CNF to develop an ionic crosslinking, where the excess of Zn^2+^ salts bridged the ENR polymer chains. The Zn^2+^ were susceptible to self-aggregation, generating ionic multiplets through strong electrostatic interaction^51,52^. The formation of clusters consisting of several ionic multiplets restricted the chain mobility of adjacent ENR polymer chains^52^. These ion-rich domain allowed the construction of ionic cross-links in the ENR network. The proposed reaction mechanism between Zn^2+^ and ENR network was also reported in the earlier works by Xu et al.^53^ and Xu et al.^28^.

### Physical properties of gloves and acid reserves in malathion

Based on the average weight and surface areas of the gloves listed in Table 1, Nitrile 1 has the largest surface area, 17% higher than NR Latex 1. Compared to the disposable gloves (Nitrile 2 and NR latex 2), Nitrile 1 has an average of 58% extensive surface area. The small surface area of the SH glove was due to the size of the lab-scale glove used in this study which was 15 cm in length. The acid reserve obtained for the pesticide is presented in Table 2. The result was presented based on 100 mL malathion. The malathion used had an acid reserve of 0.019 g at 1% and 0.264 g at 10% solution concentration.

**Table 1:** Comparison table showing surface area, weight, and weight per unit area of different gloves.

Sample	Weight of glove (g)	Weight of 9 cm^2^ piece (g)	Area density (g/cm^2^)	Glove surface area (cm^2^)	Glove finger thickness (mm)
Nitrile 1	33.42 ± 0.31	0.28	0.031	1062	0.49 ± 0.01
Nitrile 2	2.97 ± 0.04	0.05	0.006	593	0.07 ± 0.01
NR Latex 1	25.43 ± 0.46	0.26	0.029	880	0.12 ± 0.01
NR Latex 2	5.09 ± 0.11	0.07	0.008	648	0.45 ± 0.01
SH Glove	3.53 ± 0.33	0.31	0.034	104	0.38 ± 0.02

**Table 2:** Titration results of 1% and 10% malathion and the calculated acid reserves.

Concentration of malathion						
100 mL	1% solution			10% solution		
	Average titration volume (mL)	Concentration (mol)	Acid reserve (g NaOH)	Average titration volume (mL)	Concentration (mol)	Acid reserve (g NaOH)
Malathion	4.77	0.0005	0.0190	66.23	0.0066	0.2648

### Breakthrough-time analysis

**Figure 1:**
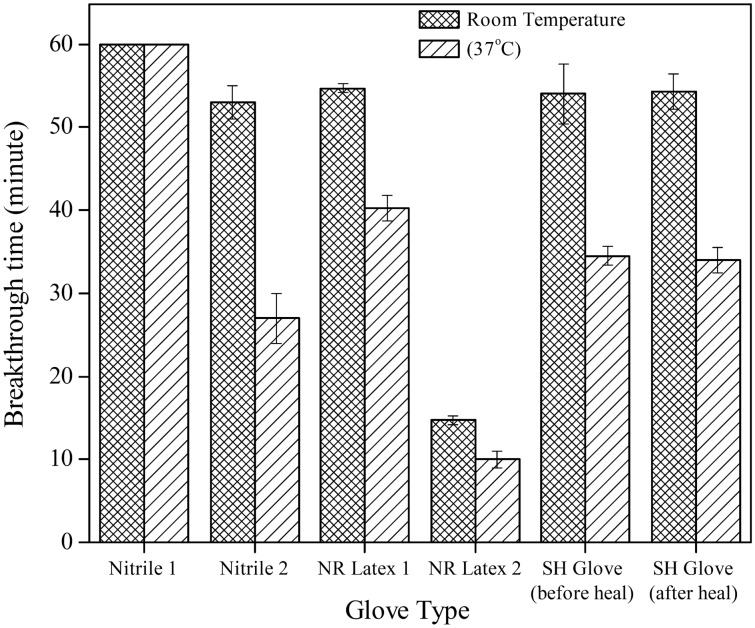
Breakthrough time of different glove type and SH glove before and after healing, evaluated at room temperature and 37 °C.

Breakthrough-time analyses conducted on the glove samples against the pesticide yielded different results for the five variants. The room temperature measurement for breakthrough time differed compared mainly to those measured at 37 °C. Breakthrough times for each glove sample between two different temperatures are presented respectively in Fig. 1 and Table 3. NR Latex 2 had the shortest breakthrough time against the pesticide compared to other samples. Nitrile 2 and NR Latex 2 recorded the highest time reduction percentage of 47% and 49%, respectively. In the permeation breakthrough time test, the Nitrile 1 glove outperformed the other tested gloves, as evidenced by the undetectable changes in the colour of the pH indicator. For NR Latex 1, the glove breakthrough times were 54.7 ± 0.6 min and 39.7 ± 1.5 min at room temperature and 37 °C, respectively, showing a time reduction of 27% at elevated temperatures.

**Table 3:** Result of average breakthrough time of the glove samples at room temperature and 37 °C. The time reduction percentage are presented in bold to show the difference.

Glove samples	Breakthrough times (min)					
	Nitrile 1	Nitrile 2	NR Latex 1	NR Latex 2	SH Glove	SH Glove
25 °C	> 60	53 ± 2	54.7 ± 0.6	14.7 ± 0.6	54 ± 3.6	53.3 ± 1.5
37 °C	> 60	27 ± 3	39.7 ± 1.5	8 ± 1	35.5 ± 2.6	34.7 ± 1.1
Time reduction (%)	Nil	**49.1**	**27.3**	**46.6**	**31.5**	**35.8**

The fabricated SH glove exhibited comparable breakthrough times as NR Latex 1, with 35%- and 37%-time reductions tested before and after self-healing, respectively, at higher temperatures. More importantly, the fabricated SH glove in this study exhibited excellent self-healing properties and characteristics which are not present in currently commercialized gloves. There were no breakthrough times recorded before and after healing for Nitrile 1, NR Latex 1, Nitrile 2, and NR Latex 2 due to the rapid leaching of the pesticide from the cut. The SH glove almost completely recovered from damages, with a 4.3% increase in time reduction between pre-and post-healing. The breakthrough time analyses conducted on the healed SH glove and NR Latex 1 samples are visualized in Supplementary Movies 1 and 2.

### Glove surface morphology

**Figure 2:**
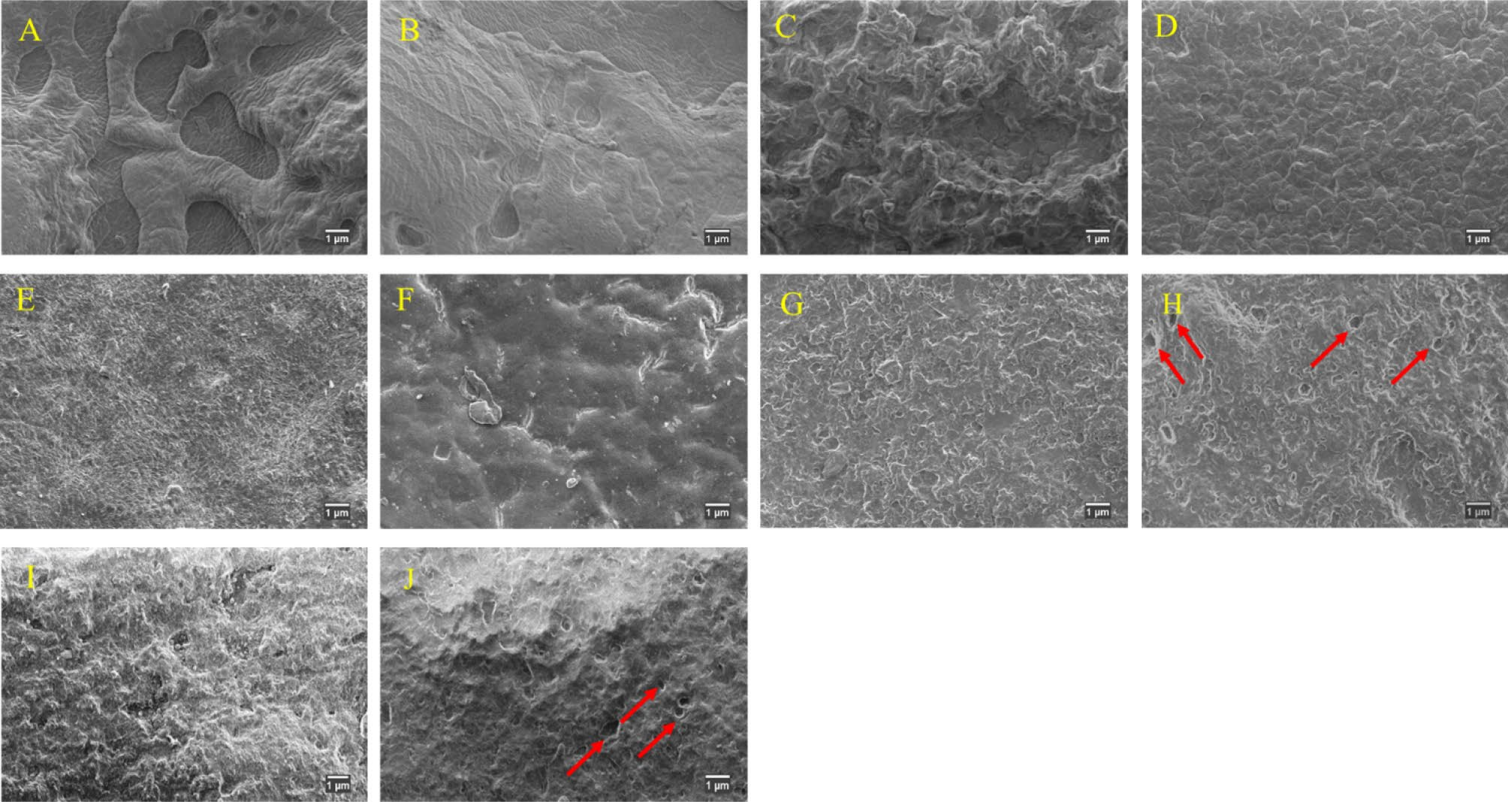
FESEM micrographs of different glove variants before and after exposure to the pesticide at 37 °C; Nitrile 1 (**A**) before, (**B**) after; NR Latex 1 (**C**) before, (**D**) after; SH glove (**E**) before, (**F**) after; Nitrile 2 (**G**) before, (**H**) after; NR Latex 2 (**I**) before, (**J**) after permeation test.

Figure 2 shows the surface morphology of gloves before and after exposure to the pesticide at 37 °C. Rough and patchy-like surface observed on the Nitrile 1 glove (Fig. 2A) become etchant after being exposed to the pesticide, as shown in Fig. 2B. There were no cracks, protrusions or holes detected in the Nitrile 1 sample after the breakthrough-time analysis of 60 min. The surface of NR Latex 1 as in Fig. 2C, appears to be rough and irregularly oriented before pesticide exposure. It changed to a smooth wavy surface with minor pinhole cracks after 55 min of pesticide exposure, as depicted in Fig. 2D. Similarly, the rough, uneven surface of the SH glove sample (Fig. 2E) transformed to a relatively smoother surface with microcracks after a 54-min average of pesticide exposure, as shown in Fig. 2F. No apparent holes were observed in both NR Latex 1 and SH glove samples when tested at the elevated temperature. As for the disposable Nitrile 2 sample, the rough patch-like morphology observed before the pesticide exposure (Fig. 2G) remains unchanged, except for a few hole-like structures, which became more distinct, as captured in Fig. 2H. The NR Latex 2 sample (Fig. 2I) was the fastest to display a distorted surface structure among all tested gloves samples, with holes and cracks observed after 15 min of pesticide exposure, as presented in Fig. 2J.

## Discussion

Choice of the gloves material types used in this experimental work was based on their ease of availability and common use in the agriculture sector. The Nitrile 1 and NR latex 1 meet standard EN 347:2016, which is the minimum acceptable for gloves used as PPE in chemical works. The acceptable quality level (AQL) information for Nitrile 2 and NR latex 2 is not available, thus it is not recommended to be used as PPE in chemical works. Despite the unsuitable condition, both disposable gloves were considered in this study due to their widespread by non-occupational handlers of agricultural pesticides^17^. The selection of malathion as a representative pesticide is based on its widespread usage in agricultural sectors and potential toxicological effects on human health^54,55^. Malathion, chemically named as diethyl 2-[(dimethoxyphosphorothioyl)sulfanyl]butanedioate, is an organophosphate pesticide used to eradicate pests of gardens, crops, ectoparasites on animals and in public health pest-elimination programs^54,56^. However, malathion exposure can potentially induce toxic effects on human health, such as disturbance in metabolism levels, lower acetylcholinesterase activities, neurotoxicity, immunotoxicity and oxidative stress in human lymphocytes^56,57^.

Malathion used in this study contained components other than the active ingredients (84%), with substantial inert ingredients. These inert ingredients act as a solvent to assist the active ingredients in penetrating plants' leaves, extending the product shelf-life, and protecting the pesticide from degradation due to sunlight exposure^58^. Our study showed that the breakthrough times for most tested glove materials, namely Nitrile 2, NR Latex 1, and SH glove, were not significantly different at a lower temperature but became more distinct at elevated temperatures. Therefore, it was hypothesized that the contained inert ingredients might have impacted the permeation of active ingredients of the pesticide through the glove material^4,17^. However, further elaborations on these effects are tricky as no detailed chemical specifications were made available for the inert ingredients used in the pesticide. In addition, the permeation effect on the gloves from the phenolphthalein indicator and NaOH added in the beaker containing water as a buffer was assumed to be negligible due to the presence of the pesticide having a greater concentration.

It has been reported that the permeation properties could be influenced by the glove material thickness^7^. Nevertheless, our results showed that the type of glove materials plays a more crucial role in affecting the chemical permeation process and rate. As shown in Table 4, Nitrile 2 thin glove (thickness: 0.06–0.08 mm) exhibited better chemical protection compared to that of thicker NR Latex 2 sample (thickness: 0.11–0.13 mm). Despite that Nitrile 1 and NR latex 1 have a similar thickness (0.44–0.50 mm), the chemical resistance performance of Nitrile 1 surpassed that of NR latex 1. This could be explained by the fact that pesticide can transfer through the glove by permeation, flowing through closures and porous materials on a molecular level driven by a chemical concentration gradient across materials. During the contact, the pesticide tends to permeate the materials with similar polarity. Among the three glove materials, nitrile with CN functional groups and ENR with OH functional groups have stronger polarity^59^. Subsequently, malathion with a relatively weaker polarity could permeate NR materials more quickly, which resulted in a shorter breakthrough time. To support this, Creta et al.^60^ reported that the NR gloves had highest permeation of chemical in comparison to nitrile gloves. Thus, this rendered the synthetic nitrile rubber a popular choice for chemical-resistant product applications^61^. The glove materials intended for future breakthrough-time analyses were suggested to have a similar thickness to eliminate the factor of material thickness when comparing chemical resistance abilities. The temperature parameter also plays a vital role in rubber glove chemical permeation. Varying temperatures from 23 to 35 °C when testing breakthrough times could reduce durations up to 30–90% depending on the type of chemicals exposed^7^. However, there are less time reductions in this study; approximately 20–50% were observed. The difference occurred due to factors such as type of glove materials, thickness, and glove manufacturer. According to Mickelsen and Hall^62^, gloves of similar features and materials, but sourced from different manufacturers, could possess breakthrough time difference up to a factor of ten. This could be contributed by the different compounding chemical formulations and manufacturing practices. Generally, reusable Nitrile 1 showed better chemical resistance abilities in both room and elevated temperatures compared to other tested glove variants.

**Table 4:** A comparison table showing different types of gloves.

Sample and colour	Product model	Producer	Finger thickness (mm)	Standard conformant	Fabrication date	Expiry date
Nitrile 1, reusable (green)	Showa 730	Showa	0.48–0.50	EN ISO 374-1:2016/Type A; EN ISO 374-5:2016	N/A	N/A
Nitrile 2, disposable (berry blue)	Shirudo 7th Sense	Cleanera	0.06–0.08	ISO 13485; ISO 9001	2020-05	2023-04
NR Latex 1, reusable (yellow)	Polycare	Top Glove	0.44–0.46	EN ISO 374-1:2016/Type B; EN ISO 374-5:2016; EN 388:2016; EU10/2011	N/A	2025-07
NR Latex 2, disposable (white)	DuraSafe	Promedictech	0.11–0.13	N/A	2020-01	2022-12
SH Glove (white)	N/A	This study	0.39–0.40	N/A	2019-06	N/A

**Figure 3:**
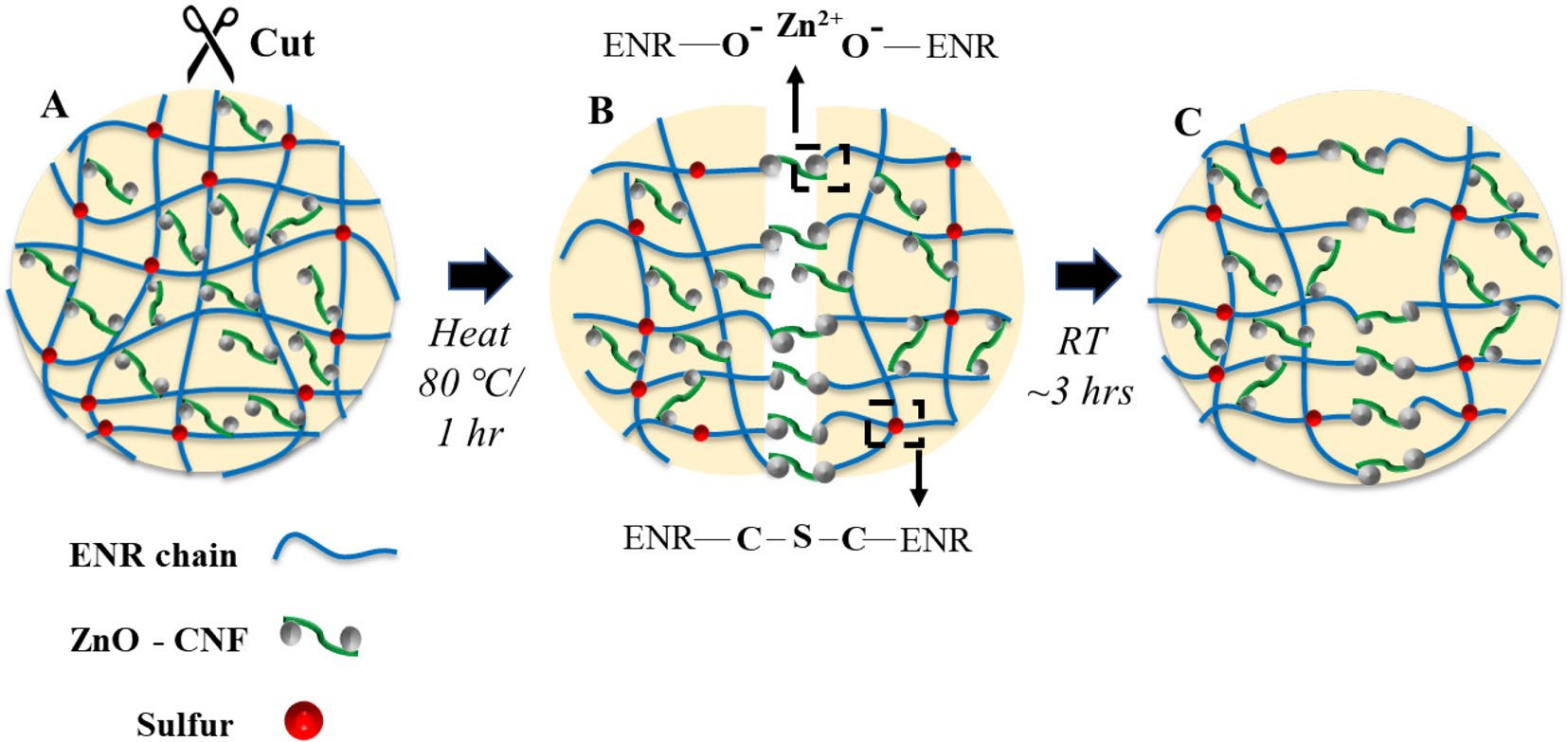
Proposed self-healing mechanism of ENR based SH glove. (**A**) Damages to the ENR matrix due to cut. (**B**) Self-repairing action. (**C**) Complete restoration of the original structural integrity after 3 h at room temperature.

Despite nitrile gloves' better chemical resistance ability, the lack of self-healing characteristics makes the glove lose its chemical barrier functionality with the presence of small tears. This is where the fabricated SH glove outshined the synthetic nitrile and other tested glove samples, which can exhibit almost complete recovery without any chemical leakage through the damaged surface, as visually supported in Supplementary Movie 1. As illustrated in Fig. 3A, the ZnO functionalized CNF acts as a cross-linker in the ENR composite chain arrangement. Under the thermal condition, the restriction of the ENR network chains diminishes, allowing the oxygenous groups along the ENR chain (ENR–O−) to interact with Zn^2+^ salt ions present on the CNF surface freely, illustrated in Fig. 3B. Finally, the reconstructed bonds between the ENR chain and Zn^2+^ ion healed when the ENR is relaxed at room temperature, as shown in Fig. 3C. The SH glove was able to undergo multiple healing cycles after repeated cut, as shown in Fig. S3A. The presence of ZnO-CNF in the ENR was investigated via FTIR and FESEM analysis, as shown in Figs. S1 and S2. The glove sample was cut at random places, approximately 50 mm, using scissors, as shown in Fig. S3B. The external stimulus of temperature (80 °C for 1 h) accelerated the self-healing process with all the cut surfaces successfully healed, as shown in Fig. S3C. Similarly, the FESEM observation in Fig. S3D,E shows clearly that the cut scar on SH glove dumbbell shape reduced significantly after the self-healing process.

Based on the FESEM result, glove materials tested displayed different degrees of morphology changes. Changes identified on the surface of glove materials are related to the chemical degradation of rubber. The degradation may subsequently lead to loss of the protective feature. The morphology result influences the breakthrough time to a certain extent. For instance, in Nitrile 1, no pesticide permeation was observed after 60 min of exposure, and no cracks, protrusions or holes were detected. The NR latex 2 glove is less resistant to the pesticide, with the fastest breakthrough time recorded with holes and minor cracks detectable on the glove sample surface. From these findings, the combination of microscopic analysis with breakthrough times provides additional knowledge and a deeper insight into pesticide exposures to rubber glove materials.

Only one pesticide was evaluated in this study, limiting the relation to other chemical classes with these selected gloves variants. The findings could be relevant to similar organophosphate pesticides, but they may not necessarily apply to different industrial chemicals such as cleaning agents and petrochemicals. As discussed earlier, malathion was selected based on its widespread usage in the agricultural sector and its potential toxic effect on human health. One possible source of error in identifying the breakthrough time of pesticides is uncertainty when recoding the end-time. The pesticide permeation was entirely determined by the color change of the indicator in the beaker containing water. Measurements on the exact time of color change of the indicators are subjective, and disparities may occur based on different individual perceptions of color. To minimize this error, the pH of the water was adjusted to enhance the initial color and improve the contrast of the indicator, which makes the endpoint observation more distinct^7^.

Continuous contact between the pesticide and gloves materials could be another possible limitation of this method as pesticide contact was not continuous in this study. However, repeated use of the gloves will induce constant contact that can affect the glove material functionality, resulting in penetration of the pesticide through compromised material in addition to the permeation. Nevertheless, only the SH glove could prevent pesticide penetration when the glove integrity is compromised among all the tested gloves. As previously mentioned, the fabricated SH gloves possess the ability to recover the loss of functionality through the self-healing process.

Despite all the possible errors and limitations, the findings clearly show that the disposable NR latex 2 glove was just suitable for handling the pesticide for a short time, less than 10 min at 37 °C. Furthermore, the chemical resistance ability of the fabricated SH glove was on par with the commercialized reusable latex glove (NR latex 1) and better than the NR latex 2 and Nitrile 2. Breakthrough time analysis remains a crucial indicator when determining the suitability of a glove in handling chemicals, although other possible factors such as compounding formulation and manufacturing process may affect the glove material penetration and permeability.

Furthermore, agricultural workers need to be equipped with adequate knowledge on the appropriate glove use for proper protection, as they are still some reported workers using disposable latex gloves while handling harmful pesticides^6,10,17^. Moreover, employers also need to be trained on the legal obligation to maintain the workers' wellbeing and understand the limited functionality of disposable gloves. Thus, an improved risk assessment on the suitability of gloves is essential, which should be conducted by employers. The breakthrough time analysis could serve as a straightforward approach to supplement the lack of data on certain glove material chemical resistance when in contact with specialized chemicals. Workers could also make use of the method without the requirement of specialized training and any additional, costly analytical equipment.

Besides the evaluation of SH gloves, the results of this study on the other tested glove variants show an urgent need to review and update the process of choosing protective gloves when handling highly concentrated pesticides. To ease the selection of the correct type of gloves, the pesticide manufacturer should make additional efforts to determine the breakthrough times of various conventional glove materials for their pesticide product. Furthermore, the pesticide manufacturer and relevant authorities should adequately assess the information provided on the SDS regarding the suitable glove type for handling the chemical. The fabricated SH glove can supplement the suggested steps in further aiding and promoting the safety of agricultural workers.

In conclusion, a novel SH glove was successfully fabricated using a conventional dipping method. The chemical resistance performance of the SH glove was assessed and compared against four different types of commercial gloves and malathion was used as a model pesticide. The results showed that the glove made with Nitrile 1 gloves exhibited greatest chemical resistance performance with no leaking detected after 1 h breakthrough time at a room temperature and 37 °C. SH gloves, Nitrile 2, and NR latex 2 presented comparable breakthrough time of ~ 53–55 min at room temperature. Based on FESEM analysis, pesticide exposure caused significant microstructural changes on the glove surfaces, as evidenced by the presence of minor cracks and etchant surface-like morphology. The self-healing test demonstrated that the developed SH glove could be self-healed via thermal treatment after being damaged by cutting. Our findings showed that SH glove with build-in self-repairing capability possesses chemical resistance properties comparable to those commercial gloves. The new self-healing features are envisaged to not only extend the working life of chemical-resistant gloves but also improve their safety and reliability index.

## Methods

### Materials

ENR latex with 50% epoxidation level and formulation reagents, as listed in Table 5, was supplied by the Malaysian Rubber Board, Sg. Buloh, Kuala Lumpur, Malaysia. The CNF used in this study was obtained from the Nanotechnology and Catalysis Research Centre, University of Malaya, Kuala Lumpur, Malaysia. Zinc acetate dihydrate (Zn(CH_3_COO)_2_·2H_2_O) and sodium hydroxide (NaOH) were purchased from Sigma Aldrich. The coagulant slurry used in this experiment consisted of a mixture of 15% calcium nitrate Ca(NO_3_)_2_ and 5% calcium carbonate (CaCO_3_), while the polymer coating was used to ease the glove stripping process from the former comprised of commercial corn starch dispersed in water. Pesticide (Wesco Malathion 84) consists of malathion—84% and 16% of inert ingredients were used in this study.

**Table 5:** Compound formulation for SH Glove fabrication.

Reagent	Amount (phr)
35% ENR latex	100.0
20% Potassium laurate (C_12_H_23_KO_2_)	0.5
10% Potassium hydroxide (KOH)	0.4
60% Sulphur	1.5
52% Zinc diethyldithiocarbamate (ZDEC)	1.0
61% Zinc oxide (ZnO)	1.3
98% Dicumyl peroxide (DCP)	0.5
35% NR latex	30.0
ZnO-CNF nanofiller	5.0

### Preparation of ZnO-CNF nanofiller

The ZnO-CNF nanofiller was fabricated according to our previously reported method^43^. In brief, 1 g dry CNF was dispersed in ultrapure water (Mili-Q^®^ Plus, Billerica, MA, USA) for 30 min using magnetic stirring. 0.44 g Zn(CH_3_COO)_2_·2H_2_O was then added to the CNF suspension. The mixture was ultrasonicated for 5 min using an ultrasonic horn system (NexTgen, Sinaptec, Lezennes, France) with a 20 kHz frequency and 100 W power rating. Then, the mixture was transferred into a three-necked refluxing flask consisting of a Liebig condenser and refluxed at 90 °C for an hour using a heating mantle. 0.1 M NaOH and 0.1 M HCl solution were used to adjust the pH of the mixture to 10. The mixture was then continuously stirred for 30 min at 500 rpm and subsequently subjected to the ultrasonication cycle for another 5 min. The obtained ZnO-CNF nanofiller was rinsed with ethanol and washed with ultrapure water for multiple cycles to remove any residual chemicals. The wet ZnO-CNF slurry was immediately added to the ENR latex mixture.

### Preparation of SH glove

**Figure 4:**
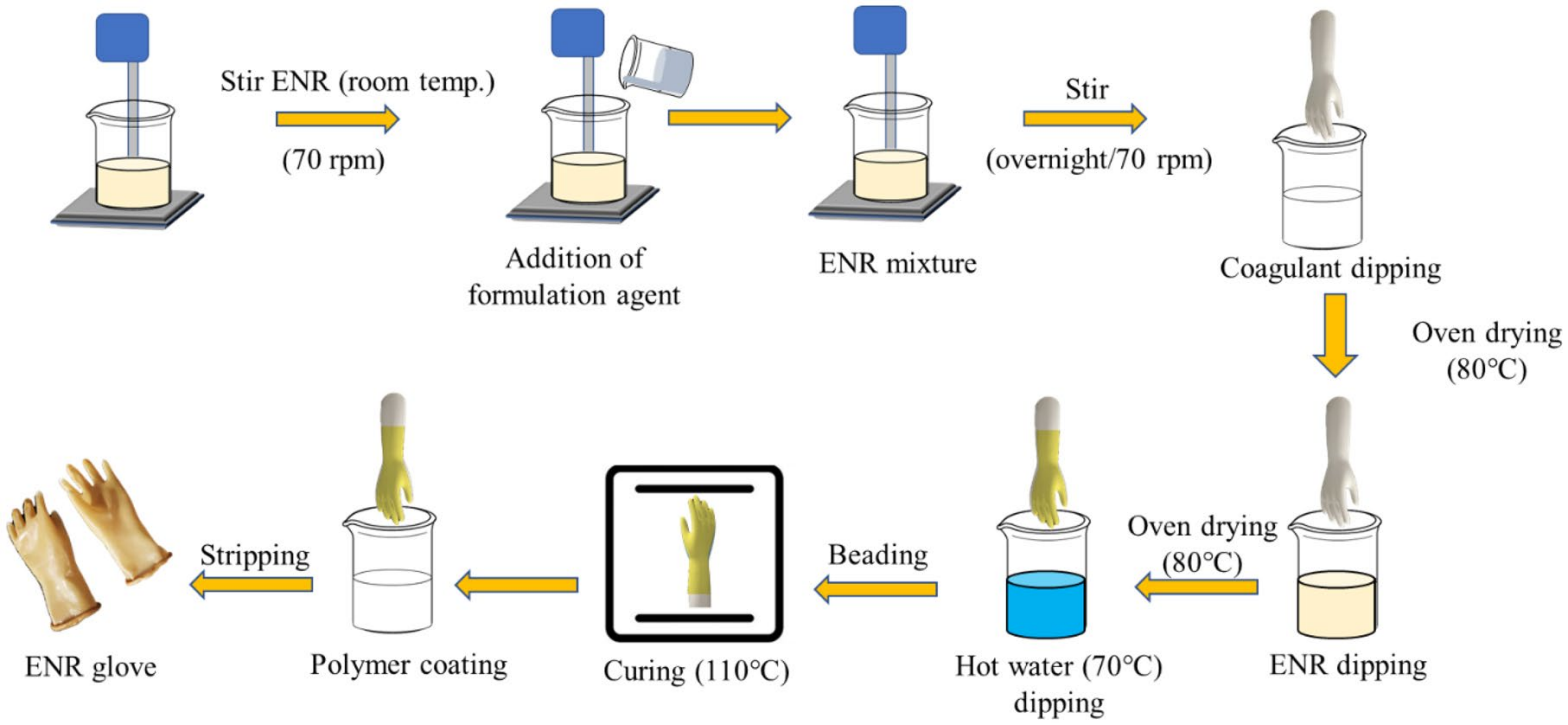
Fabrication process of SH glove using a ceramic hand former.

A mini hand ceramic former with a length of 15 cm was used to fabricate the self-healable glove. The latex dipping formulation was prepared following the recipe shown in Table 5. The total ENR latex used was 400 g by weight. The hand former was thoroughly cleaned with acid (1 M HCl), alkali (1 M NaOH) and washed with water to remove any deposited dirt. After drying, the cleaned former was dipped into the coagulant slurry, dwelled for 15–20 s. The former was then dried in an oven at 80 °C for 10 min. The former was then dipped into the prepared ENR/ZnO-CNF latex formulation for 30–40 s. This was followed by slow manual rotation to ensure a uniform latex coating around the former. The former was then dried again in the oven at 80 °C for 30 min. The coagulant and ENR/ZnO-CNF latex dipping process with drying was repeated for an additional two cycles. After the third cycle, the former was subjected to water leaching at a temperature of 70 °C for 1 min and later proceeded to glove cuff beading. Subsequently, the former was dried at 110 °C for 20–30 min. The cured glove was then dipped in the polymer suspension for 5 s. Finally, the glove was stripped from the hand former and left to dry at room temperature. The glove fabrication process is illustrated in Figure 4.

### Commercial glove used for comparison

Gloves made of two different materials, namely synthetic nitrile rubber and NR, were chosen for comparison in this research based on the recommendation of these materials in several product safety data sheets. The reusable nitrile (Showa 730) and NR latex glove (Polycare, Top Glove) were labeled suitable for chemical protection following standard EN ISO 374:2016. Two additional types of gloves, namely disposable nitrile (Shirudo 7th Sense, Cleanera) and disposable NR latex (Durasafe, Promedictech) gloves were also considered, which were designed for laboratory and medical examination applications. Although not suitable for extreme chemical handling, disposable gloves were included for comparison as agricultural workers widely used these gloves in their daily activities^10,17^. The list of glove variants used as a benchmark could be found in Table 4.

### Determination of weight and surface area of gloves

Medium-sized gloves were used in the study except for the fabricated self-healing glove, which was 15 cm in length. Three gloves were selected from each variant and were weighed using a standard laboratory weighing scale (HR-250AZ, A&D Company, Japan). The average weight of each set was determined. Three sample pieces of 9 cm^2^ were cut from various parts of the glove and weighed. The average weight of these pieces was also determined. The weight per unit area of glove material was calculated based on the average weight of each material type. The total surface area of the glove was used as the basis for size comparison. The total surface area was calculated based on the weight per unit area and the average weight of the gloves^7^.

### Acid reserve determination

Acid reserves were investigated for 10% and 1% solution concentration of malathion. 100 mL of 1% and 10% malathion was titrated with 0.1 M NaOH, while the pH value was measured using a pH meter (Mettler Toledo, FiveEasyPlus, Greifensee, Switzerland). The titration process was stopped when the pH value was observed to stabilize. The acid reserve was determined from the titratable acid in malathion and was expressed in grams of NaOH.

### Permeation breakthrough-time analysis

The breakthrough-time test in the study was conducted according to the method reported previously^7^. Firstly, 10 mL of malathion was poured onto the glove sample's cut finger piece, which was inverted inside out. Next, the fingers containing malathion were immersed in 100 mL deionized water with phenolphthalein as a pH indicator. The pH level of the water was regulated between 8.3 to 8.4 using 0.1 M NaOH. pH regulation of the water improves the clarity color changes when the acidic solution is mixed with water, which enables clear endpoint determination and precise recording of the end time. The first color change of the indicator indicates the chemical breakthrough from the glove sample, thus indicating the presence of malathion in the water. Breakthrough-time analysis was conducted with the water at room temperature (25 °C) and body temperature (37 °C). Due to the miniature size of the SH glove, the whole fabricated sample was used for the breakthrough-time analysis.

### Self-healing test

For the self-healing study, the finger region of the gloves of approximately 50 mm in length was cut using scissors. The samples were then subjected to similar breakthrough-time analysis. Then, the glove is removed from the beaker and washed thoroughly with running water to remove malathion. The washed samples were then dried partially using an air blower to remove excess water. The exposed cut surfaces were put in contact with each other and heated in an oven at 80 °C for 1 h. The samples were then removed and left to cool to room temperature for approximately 3 h to complete the self-healing process. The breakthrough-time analysis was repeated on the healed glove samples.

### Morphological observation

The glove finger samples’ surface structures before and after exposure to malathion were investigated using a Field Emission Scanning Electron Microscope (FESEM, Oxford-Horiba Inca XMax50, Hitachi SU8010, Tokyo, Japan).

## Supplementary Information

Supplementary Information.

Supplementary Video 1.

Supplementary Video 2.
