# Supplementary material for: Assessing the suitability of self-healing rubber glove for safe handling of pesticides
Source: Sci Rep. 2022 Mar 11;12:4275. doi: 10.1038/s41598-022-08129-9 (PMC8917143; doi:10.1038/s41598-022-08129-9)
Supplement: Supplementary file 1 — Supplementary Information. [file 41598_2022_8129_MOESM1_ESM.docx]

**Supplementary Information**

**Assessing the Suitability of Self-healing Rubber Glove for Safe Handling of Pesticides**

Janarthanan Supramaniam^1^, Darren Yi Sern Low^1^, See Kiat Wong^1^, Bey Hing Goh^2,3^, Bey Fen Leo^4,5,*^ and Siah Ying Tang^1,6,*^

^1^Chemical Engineering Discipline, School of Engineering, Monash University Malaysia, 47500 Bandar Sunway, Selangor Darul Ehsan, Malaysia.

^2^Biofunctional Molecule Exploratory Research Group, School of Pharmacy, Monash University Malaysia, 47500 Bandar Sunway, Selangor Darul Ehsan, Malaysia.

^3^College of Pharmaceutical Sciences, Zhejiang University, Hangzhou, 310058 Zhejiang Province, China.

^4^ Nanotechnology and Catalysis Research Centre, University of Malaya, 50603 Kuala Lumpur, Malaysia.

^5^Faculty of Medicine, University of Malaya, 50603 Kuala Lumpur, Malaysia.

^6^Advanced Engineering Platform, School of Engineering, Monash University Malaysia, 47500 Bandar Sunway, Selangor Darul Ehsan, Malaysia.

*Corresponding Author 1: Bey Fen Leo. Email: *beyfenleo@um.edu.my

*Corresponding Author 2: Siah Ying Tang. Email: patrick.tang@monash.edu

Fourier Transform Infrared Spectroscopy (FTIR)

The presence of ZnO-CNF and ENR matrix was analyzed using a Fourier-Transform infrared spectrometer (FTIR, Varian 600-IR series, Varian, Mulgrave, Australia) at a range of 400 – 4000 cm^-1^ over 32 scans. The original SH glove (ENR/ZnO-CNF) specimen and thermally healed specimen were examined using the FTIR to determine the effect of heat treatment on the ENR/ZnO-CNF chemical structure.

Tensile Strength and Self-Healing Investigation

The original ENR/ZnO-CNF specimen’s ultimate tensile strength and strains were determined using a universal testing machine (INSTRON 5966, UK) at a crosshead speed of 500 mm per minute. All reported tensile measurements were triplicated.

The self-healing ability of the prepared specimens was tested according to the reported methods^1-3^. The dumbbell-shaped specimens were cut in the middle with scissors, and the surfaces of the two separated pieces were then brought into close contact with each other. Then, the cut specimens were placed in an oven at 80℃ for 1 hr. The thermally treated specimens were removed from the oven and placed at room temperature for 3 hrs. The healed specimens were subjected to stress-strain tests at similar crosshead speeds, and all measurements were triplicated. The degree of recovery or termed as self-healing performance, $\text{η,}$ was quantified using **Equation (1)**^2^.

$\text{η }\left( \text{\%} \right)\text{ =}\frac{\text{E}_{\text{healed}}}{\text{E}_{\text{original}}}\text{ × 100\%}$ **(1)**

where E_original_ refers to the tensile energy exhibited by the uncut specimens and E_healed_ refers to the tensile strength of healed specimens.

Additionally, the dumbbell shape specimens before cut and post-healing were subjected to morphology analysis using FESEM at 5 kV.

Chemical Interaction of ZnO-CNF with ENR Matrix

As shown in **Figure S1**, the FTIR analysis was conducted to confirm the presence of ZnO-CNF in the ENR composite. For this purpose, a neat ENR was fabricated using the formulation **Table 1** in the main manuscript, without ZnO-CNF nanofiller.

**
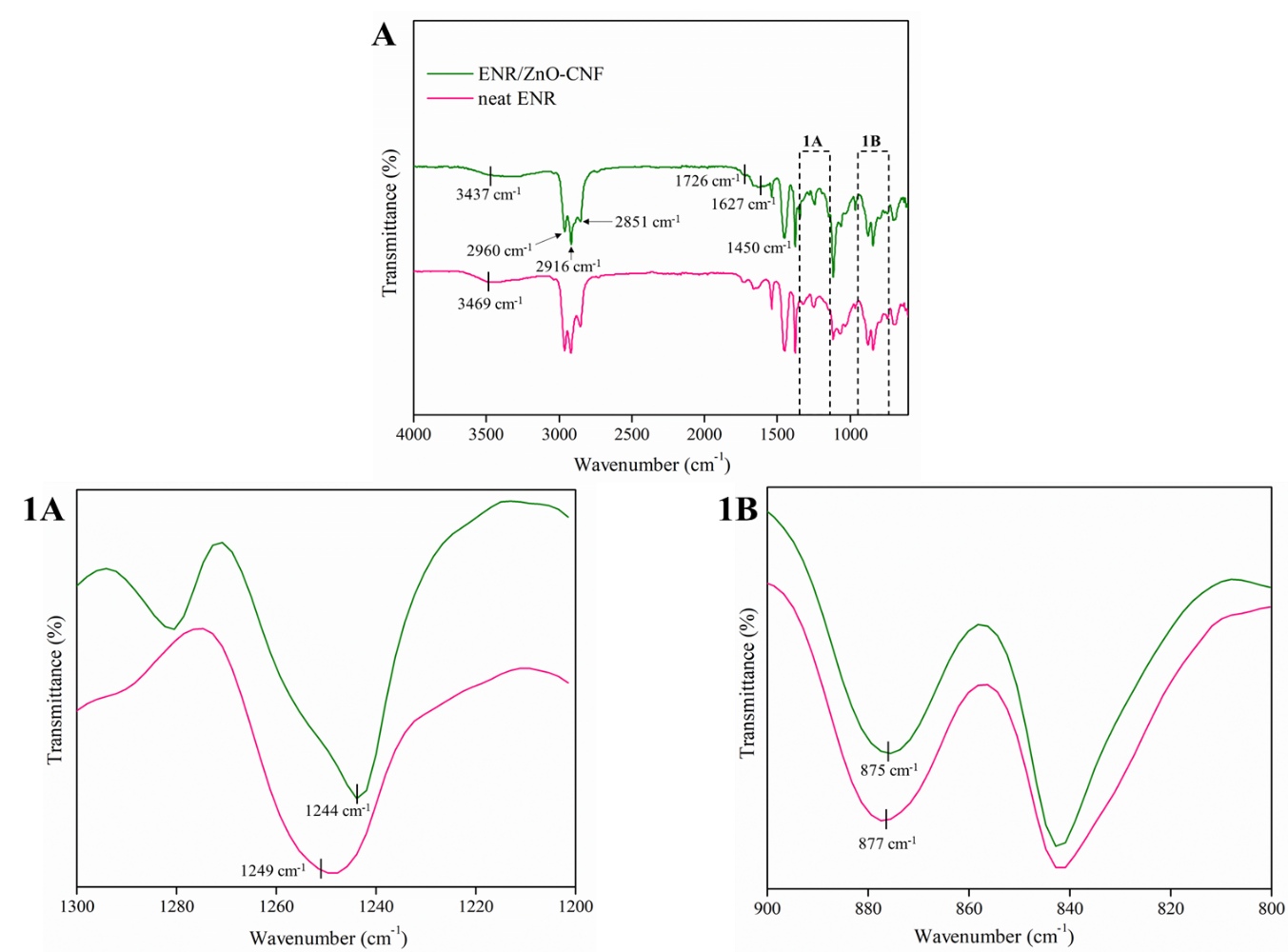
**

**Figure S1:** (A) FTIR spectra of neat ENR and ENR/ZnO-CNF; (1A) focused FTIR spectra between 1200 – 1300 cm^-1^; (1B) focused FTIR spectra between 800 – 900 cm^-1^.

The broad peak in the range of 3200-3500 cm^-1^ is related to hydroxyl groups ^2^. The spectrum identified for the ENR/ZnO-CNF at 2960 cm^-1^ and 1450 cm^-1^ was attributed to methyl asymmetrical stretching deformation vibrations^4^. The spectrum assigned to the methylene asymmetrical stretching and the symmetrical stretching vibrations were determined at 2916 cm^-1^ and 2851 cm^-1^ for the ENR composite^5^. The peak detected at 1627 cm^-1^ was attributed to the C=C alkene functional group and these bands were associated with ring-opening products^6^. An enlarged graph of the FTIR spectra is presented in Figure **S1 (1A)** and (**1B)** to interpret the peak changes better. The interaction between a filler and ENR can be supported by changes in the spectra of 1249 – 1260 cm^-1^ and 862 – 875 cm^-1^ peaks assigned to the epoxy group of ENR^7-9^. The vibrational peak attributed to the symmetrical stretching and asymmetrical stretching of the epoxy group in neat ENR were represented by the spectra identified at 877 cm^-1^ and 1249 cm^-1 10^. However, with the introduction of ZnO-CNF nanofiller, the vibrational mode shifted to a lower wavenumber of 1244 cm^-1^ and 836 cm^-1^ for ENR/ZnO-CNF composite. Furthermore, the peak at 3469 cm^-1^ attributed to the hydroxyl group in neat ENR shifted to 3437 cm^-1^ for ENR/ZnO-CNF composite. Similarly, Cao, et al. ^2^ reported the addition of cellulosic materials decreased the peak intensity of ENR hydroxyl groups detected at 3450 cm^-1^.

Cross-section analysis of SH Glove

The morphological analysis conducted on the cross-section of ENR/ZnO-CNF using the FESEM was shown in **Figure S2**. The presence of ZnO-CNF can be identified from the random large pull-outs of fibrous structure, as shown in **Figures S2(A), (B**) and **(C)**. Additional holes shown in **Figure S2(B)** (blue arrows) indicated the traces of ZnO-CNF pull-outs. The morphology findings in **Figure S2** provide additional support to the FTIR analysis on the presence of ZnO-CNF nanofiller in SH glove.


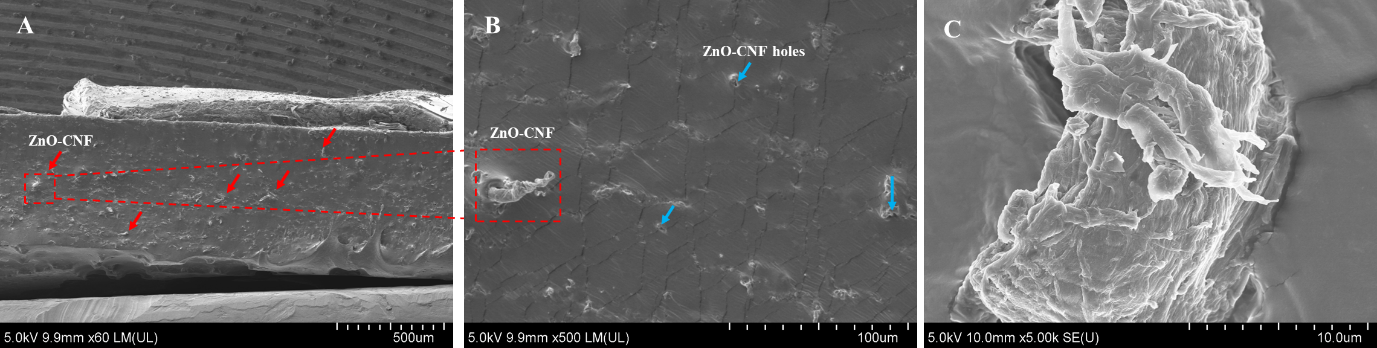


**Figure S2:** (A) FESEM images of ENR/ZnO-CNF cross-section (red arrows show ZnO-CNF); (B) enlarged image ZnO-CNF and ZnO-CNF pull-outs holes; (C) enlarged image of ZnO-CNF fibrous structure.

Tensile Strength and Self-Healing Properties of SH Glove

The ultimate tensile strength of the SH glove is 5.27 ± 0.37 MPa. The embedment of ZnO in the CNF matrix improved the tensile strength of the ENR composite. This could be ascribed to the common role of ZnO as a crosslinker in rubber processing and as an activator in vulcanization processes^11-13^. The aggregation of Zn^2+^ salts consisting of ionic pairs forms ionic domains with high moduli which is responsible for the increase in tensile strength of the rubber materials^14^. In the present work, we have proved that the addition of a hybrid nanofiller based on ZnO-CNF led to substantial improvement in tensile strength in the self-healing rubber, up to 5.27 ± 0.37 MPa compared to many existing literatures works with tensile strength approximately between 1.2 MPa – 4.0 MPa^2,8,15-18^.


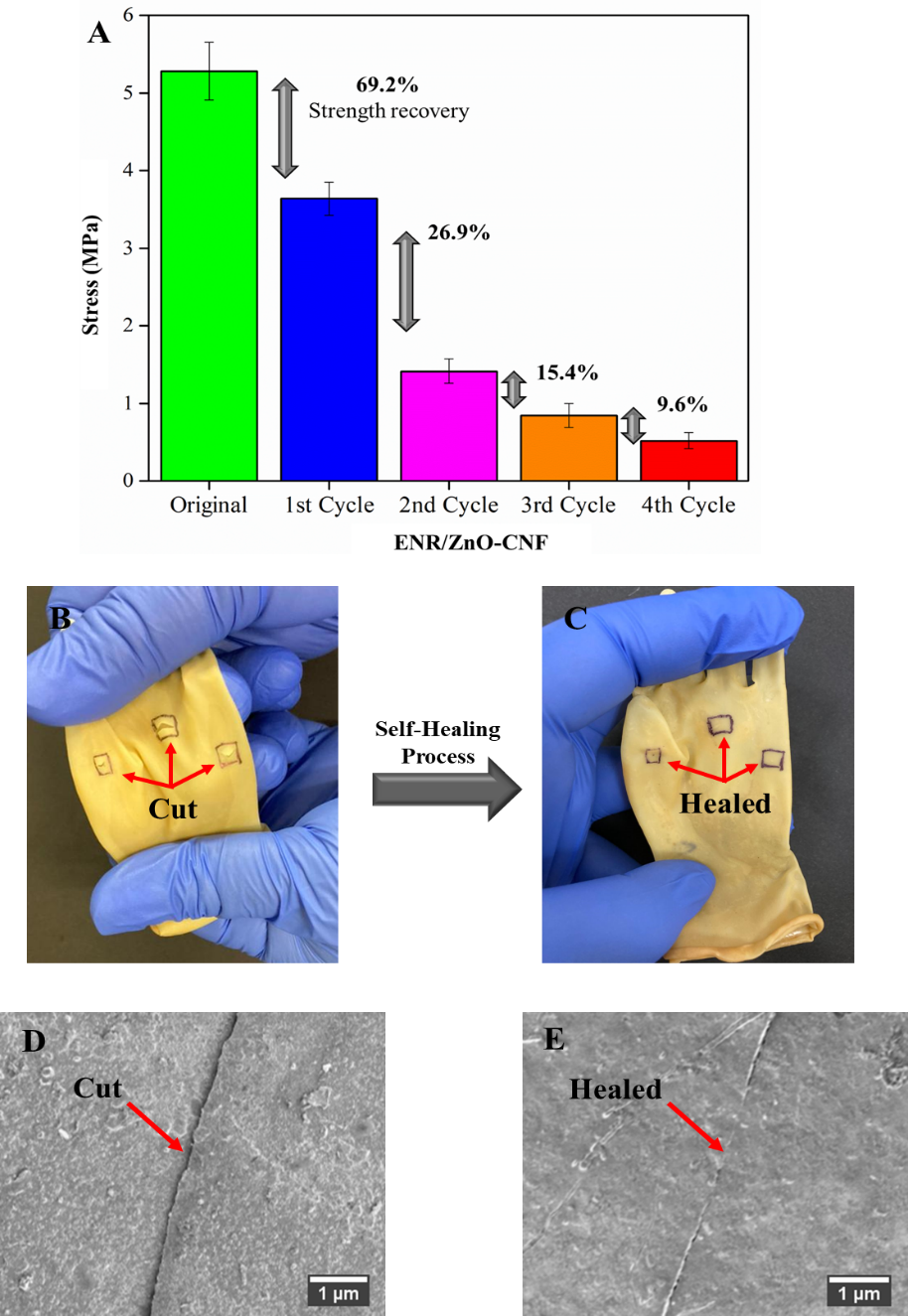


**Figure S3:** (A) Tensile strength, repeated healing cycle and self-healing performance of SH glove; Image of cut and healed specimens (B) Cut SH glove specimen; (C) Healed SH glove specimen; (D) FESEM observation of cut specimen; (E) FESEM observation of healed specimen

Besides the breakthrough time test, self-healing characteristics were tested by cutting the SH glove’s dumbbell samples into two pieces. Subsequently, the surfaces of the two cut pieces were brought into close contact and allowed to self-heal 1 hour at 80℃ and allowed to cool down at room temperature for 3 hours. As shown in **Figure S3(A)**, the strength recovery increased up to 3.63 ± 0.21 MPa, which was 69.2% of its original value of 5.27± 0.37 MPa. When the cut sample is heated at 80 ⁰C, the surviving reversible bonds in the ENR composite matrix break at elevated temperatures^19^. The restriction of ENR network chains diminishes, thus shortening the time for reconstruction. When the ENR chains were relaxed, the reconstructed reversible bonds stabilized upon cooling at room temperature, leading to enhanced self-healing properties.

The repeated self-healing cycle findings were shown in **Figure S3(A)**, where the tensile strength of SH glove composite decreased to 1.41 ± 0.16 MPa in the second healing cycle. After the third healing cycle, tensile strength recoveries were at 0.84 ± 0.15 MPa, respectively. Finally, after the fourth healing cycle, the tensile strength was maintained at 0.52 ± 0.1 MPa. The recovery of reversible bonds only occurs on surfaces in contact; hence fractured surfaces must be in maximum contact for optimal self-healing process^18^. It is obvious that the unhealed parts will easily lead to stress concentration points and fracture during stretching, causing a decrease in strength recovery during repeated healing cycles. It is noteworthy the SH glove fabricated in this was able self-heal multiple cycles after repeated damage. The image as supplemented in **Figure S3(B)** shows the SH glove were cut randomly at three places (marked with squares). A noticeable reduction in cut scar visibility and shrinkage of the scar was observed in **Figure S3(C)** post thermal treatment. Similarly, the FESEM observation in **Figure S3(D)** and **(E)** shows clearly that the cut scar on SH glove dumbbell shape reduced significantly after the self-healing process.

**References**

**1.** Das, A. *et al.* Ionic Modification Turns Commercial Rubber into a Self-Healing Material. *ACS Appl. Mater. Interfaces* **7**, 20623-20630; https://doi.org/10.1021/acsami.5b05041 (2015).

**2.** Cao, L., Yuan, D., Xu, C. & Chen, Y. Biobased, self-healable, high strength rubber with tunicate cellulose nanocrystals. *Nanoscale* **9**, 15696-15706; https://doi.org/10.1039/c7nr05011a (2017).

**3.** Xu, C., Cao, L., Lin, B., Liang, X. & Chen, Y. Design of Self-Healing Supramolecular Rubbers by Introducing Ionic Cross-Links into Natural Rubber via a Controlled Vulcanization. *ACS Appl. Mater. Interfaces* **8**, 17728-17737; https://doi.org/10.1021/acsami.6b05941 (2016).

**4.** Amornchaiyapitak, C., Taweepreda, W. & Tangboriboonrat, P. Modification of epoxidised natural rubber film surface by polymerisation of methyl methacrylate. *Eur. Polym. J.* **44**, 1782-1788; https://doi.org/10.1016/j.eurpolymj.2008.03.002 (2008).

**5.** Xu, T., Jia, Z., Luo, Y., Jia, D. & Peng, Z. Interfacial interaction between the epoxidized natural rubber and silica in natural rubber/silica composites. *Appl. Surf. Sci.* **328**, 306-313; https://doi.org/10.1016/j.apsusc.2014.12.029 (2015).

**6.** Van Zyl, A. J. P., Graef, S. M., Sanderson, R. D., Klumperman, B. & Pasch, H. Monitoring the grafting of epoxidized natural rubber by size-exclusion chromatography coupled to FTIR spectroscopy. *J. Appl. Polym. Sci.* **88**, 2539-2549; https://doi.org/10.1002/app.12061 (2003).

**7.** Li, J. *et al.* Strong, tough and healable elastomer nanocomposites enabled by a hydrogen-bonded supramolecular network. *Compos. Commun.* **22**, 100530; https://doi.org/10.1016/j.coco.2020.100530 (2020).

**8.** Nie, J. *et al.* Strengthened, Self-Healing, and Conductive ENR-Based Composites Based on Multiple Hydrogen Bonding Interactions. *ACS Sustain. Chem. Eng.* **8**, 13724-13733; https://doi.org/10.1021/acssuschemeng.0c04136 (2020).

**9.** Cao, L., Huang, J. & Chen, Y. Dual Cross-linked Epoxidized Natural Rubber Reinforced by Tunicate Cellulose Nanocrystals with Improved Strength and Extensibility. *ACS Sustain. Chem. Eng.* **6**, 14802-14811; https://doi.org/10.1021/acssuschemeng.8b03331 (2018).

**10.** Rahman, A., Sartore, L., Bignotti, F. & Di Landro, L. Autonomic self-healing in epoxidized natural rubber. *ACS Appl. Mater. Interfaces* **5**, 1494-1502; https://doi.org/10.1021/am303015e (2013).

**11.** Panampilly, B. & Thomas, S. Nano ZnO as cure activator and reinforcing filler in natural rubber. *Polym. Eng. Sci.* **53**, 1337-1346; https://doi.org/10.1002/pen.23383 (2013).

**12.** Li, Y., Sun, H., Zhang, Y., Xu, M. & Shi, S. Q. The three-dimensional heterostructure synthesis of ZnO/cellulosic fibers and its application for rubber composites. *Comp. Sci. Tech.* **177**, 10-17; https://doi.org/10.1016/j.compscitech.2019.04.012 (2019).

**13.** Przybyszewsa, M. & Zaborski, M. The effect of zinc oxide nanoparticle morphology on activity in crosslinking of carboxylated nitrile elastomer. *eXPRESS Polym. Lett.* **3**, 542-552; https://doi.org/10.3144/expresspolymlett.2009.68 (2009).

**14.** Xu, C. *et al.* Design of “Zn2+ Salt-Bondings” Cross-Linked Carboxylated Styrene Butadiene Rubber with Reprocessing and Recycling Ability via Rearrangements of Ionic Cross-Linkings. *ACS Sustain. Chem. Eng.* **4**, 6981-6990; https://doi.org/10.1021/acssuschemeng.6b01897 (2016).

**15.** Chen, Y. *et al.* Covalently Cross-Linked Elastomers with Self-Healing and Malleable Abilities Enabled by Boronic Ester Bonds. *ACS Appl. Mater. Interfaces* **10**, 24224-24231; https://doi.org/10.1021/acsami.8b09863 (2018).

**16.** Wang, D. *et al.* Intelligent rubber with tailored properties for self-healing and shape memory. *J. Mater. Chem. A* **3**, 12864-12872; https://doi.org/10.1039/C5TA01915J (2015).

**17.** Xu, C., Nie, J., Wu, W., Fu, L. & Lin, B. Design of self-healable supramolecular hybrid network based on carboxylated styrene butadiene rubber and nano-chitosan. *Carbohydr. Polym.* **205**, 410-419; https://doi.org/10.1016/j.carbpol.2018.10.080 (2019).

**18.** Nie, J., Mou, W., Ding, J. & Chen, Y. Bio-based epoxidized natural rubber/chitin nanocrystals composites: Self-healing and enhanced mechanical properties. *Compos. B. Eng,* **172**, 152-160; https://doi.org/10.1016/j.compositesb.2019.04.035 (2019).

**19.** Liu, J. *et al.* An advanced elastomer with an unprecedented combination of excellent mechanical properties and high self-healing capability. *J. Mater. Chem. A* **5**, 25660-25671; https://doi.org/10.1039/C7TA08255J (2017).
